# Analyzing anomalous events in passageways with high-frequency ship signals

**DOI:** 10.1371/journal.pone.0320129

**Published:** 2025-04-01

**Authors:** Cherryl Chico, Macario O. II Cordel, Mahinthan Joseph Mariasingham, Elaine S. Tan

**Affiliations:** Data Division, Economic Research and Development Impact Department, Asian Development Bank, Mandaluyong, Philippines; Vietnam Maritime University, VIET NAM

## Abstract

Passageways are critical gateways in maritime trade, providing efficient routes for global commerce. This means that disruptions such as natural disasters or human error at these crucial points can significantly impact numerous economies across various facets of trade. Thus, real-time monitoring of such events and their impact on different vessel types is crucial for developing timely mitigation strategies, policies, and penalties. This paper proposes a novel framework for analyzing anomalous events in passageways using Automated Information System (AIS). This framework enables monitoring of affected vessels and quantifies the impact in terms of the number of vessels, dwell and idle times, and the emergence of new routes. To demonstrate its potential, we applied the proposed framework to the Suez Canal blockage in March 2021. Results revealed distinct phases during the event, with each phase impacting average dwell times. Additionally, the analysis identified the emergence of passageway event-driven routes utilized by vessels to bypass the blockage. These findings highlight the ability of the proposed framework to capture previously obscured information compared to existing indicators and port analysis methods. To further demonstrate its generalizability, the methodology was also applied to two other passageways: the Bosporus Strait, following the policy implemented in February 2022 due to the Russian invasion of Ukraine, and the Bab el-Mandeb Strait, following the disruption caused by the Houthi attacks beginning in the last quarter of 2023.

## Introduction

Passageways as the lifeblood of global trade play an unparalleled role in facilitating the movement of goods across continents. A staggering 80% of global trade volume relies on ships [1], making these trade gateways, critical arteries for our interconnected world. Any disruption in these passageways can have significant ramifications, leading to increased costs and compromised maritime connectivity. As highlighted by UNCTAD in their report [1], the impact can be far-reaching, with consequences cascading across different industries and regions. For example in Panama Canal, where even minor restrictions can force ships to take alternative routes, adding days to transit times and incurring substantial financial losses. Furthermore, passageways are inherently susceptible to external shocks, from natural disasters to human errors, making them one of the most uncertain components within the global supply chain.

To effectively assess the impact of such events and design timely solutions, real-time maritime indicators that reflect the actual situation on the ground are needed. While existing Automated Information System (AIS)-based indicators like arrival time, berth idle time, and cargo operations time provide valuable insights, they often suffer from limitations. Annual reports like the World Bank’s Container Port Performance Index [2], while comprehensive, lack the real-time element crucial for immediate response. Conversely, more frequent data sources like the IMF’s port calls count [3–5] are optimized for port activities and may not be suitable for analysis of anomalous events in passageways.

Several studies have investigated methods in using AIS data for detecting anomalous maritime events. Researchers [6–9], have explored the utilization of AIS data to model typical motion patterns of ships and detect abnormal behavior. These studies emphasize the effectiveness of spatial and thematic attributes in detecting abnormal ship trajectories and predicting ship behavior rather than abnormal trade gateway events.

Another study by Harun-Al-Rashid and his colleagues [10] investigates anomalies in maritime traffic using AIS and multisensory satellite imagery. Their research reveals insights into ship congestion through satellite images and sudden speed changes through AIS data during the Suez Canal obstruction, showcasing the potential of combining AIS and satellite data for anomaly detection. However, the complexity of integrating AIS with satellite imagery poses challenges in data processing and analysis, limiting the scalability of such approaches.

Our work proposes an AIS-only-based methodology for investigating anomalous events at passageways. The following are the contributions of this work:

- Our proposed AIS-based framework offers a more comprehensive picture of anomalous events in passageways, enabling real-time monitoring of their development across the entire location of interest until resolution. Demonstrated by its application to the Suez Canal blockage in March 2021, our approach using AIS-derived indicators (*daily vessel count*, *daily median dwell and idle times*, and *passageway event-driven routes*) revealed distinct phases during the event, their impact on dwell time, and the emergence of reroutes, which are information concealed by current indicators and port analysis methodologies.
- Our study introduces a novel scheme for monitoring transiting vessels within passageways, addressing limitations of existing approaches that solely rely on entry and exit points. Empirical evaluation of the Suez Canal revealed that our scheme, incorporating data collection redundancy, improves vessel detection accuracy by approximately 10% compared to other AIS-based detection methods [11,12].
- Our solution is generalizable and scalable to a wide range of passageway disruptions. We demonstrated this by analyzing two other disruptions in the Bosporus and Bab el-Mandeb Straits. Our open-source methodology can be deployed by readers to study similar anomalous events, making our methodology accessible and adaptable for broader applications. The code and data are available at: https://github.com/cherrylchico/ais_passageways/tree/main.

## Related works

The high-frequency nature of AIS signals allows for detailed data collection, which has been utilized to curate reliable vessel behavior [13], and activities in ports [14,15] and passageways [16,17]. Various studies have developed algorithms for detecting abnormal ship behavior in open waters using AIS data. Notably, Shi et al. [6], Gao et al. [7], Singh and Heymann [8], and Ristic [18] employed position reports and voyage data from AIS to monitor and identify anomalies in vessel movements. Shu et al. [19] examined how external factors such as wind and visibility influence the speed, course, and path of vessels. In dynamic route planning, Andersson and Ivehammar [20] introduced an AIS-derived cost indicator for cost-benefit analysis. For inland waterways, Dobbins and Langsdon [13] analyzed trip information of vessels on the Mississippi and Ohio rivers, while Xiao et al. [21] developed a simulation model to prevent collisions using AIS data on location, speed, and course.

In monitoring port and bay activities, Coomber and fellow researchers [14] utilized AIS data to track maritime traffic in ports and marine protected areas. Subsequently, Jia et al. [22] introduced an AIS-based indicator to assess the connectivity of Norwegian ports using data from multiple vessel types over a seven-year period. More recently, AIS signals have been employed to measure port traffic and estimate economic activity. Kang and his team [23] derived indices for traffic speed, rate, and dwell time at ports. Arslanap et al. [3] and Cerdeiro et al. [4] used indicators to gauge trade volume in ports. Harun-Al-Rashid et al. [21] extended port studies by detecting maritime traffic anomalies, particularly in the Suez Canal, using AIS data to monitor vessel speed and course and satellite data to monitor congestion.

Our work presents a framework for analyzing anomalous events in passageways using AIS data exclusively. We develop a methodology that is validated with ground truth to estimate congestion-related indicators within passageways, i.e. vessel counts, their dwell and idle times within the passageway, and rerouting behavior. Such indicators are critical to maritime authorities managing passageway disruptions, and researchers studying vessel and trade movements. Table 1 summarizes the related works that utilize AIS-based indicators, highlighting the differences between our work and others.

**Table 1:** Summary of related works that use AIS data and AIS-derived indicators. Our work differs from other works focusing on passageway analysis using AIS-only-based indicators.

Studies	Focus	AIS data/AIS-derived indicators
[6–8,18]	open water	GPS location, speed, static and voyage data
[20]	open water	Total cost for running a ship
[13,19,21]	open water	GPS location, speed
[23]	inland waterways	Traffic speed index, traffic rate index, dwell time at port index
[14,22]	protected area, ports	GPS location, speed, static and voyage data
[3]	ports	Cargo number indicator; cargo load indicator
[4,5]	ports	Predicted port call, volume of trade
[10]	canal	GPS location, speed, ship trajectory, ship distribution with satellite data info
[16,17]	passageway	Dwell time, vessel counts
Present study	passageways	Dwell time, Idle time, Vessel counts, Reroutes

## Data and study area

This study utilizes AIS data accessed through the UN Global Platform or UNGP (https:// unstats.un.org/bigdata/un-global-platform.cshtml). The AIS data, provided by Spire (https://spire.com/) [24], have varying reporting intervals depending on the vessel. Typically, AIS transponders send location reports every 2 to 10 seconds when a vessel is moving and every 3 minutes when stationary [25]. For terrestrially-received AIS data, Spire reduces these to one data point per 5-minute interval to manage data volume while retaining key information. The UNGP then preprocesses the AIS data to remove duplicate records, correct errors, and handle missing data, ensuring the dataset’s accuracy and reliability. Additionally, the UNGP defines specific geographic boundaries or regions of interest, allowing AIS data to be filtered to exclude irrelevant information. For example, data from active ships within specific geographic boundaries are retained during a data query. The UNGP also addresses variations in data reporting frequency and incorrect Maritime Mobile Service Identities (MMSI) formats, ensuring conformance to AIS specifications [24]. The preprocessed data are publicly available through the UNGP.

For this work, further processing the AIS data involved monitoring vessels, focusing only on trade-related vessels, *e.g.* container ships, tankers, and bulk carriers, that pass through at least two Points of Interest (POIs). Trade-related vessels that do not pass through two POIs are considered to be visiting a port rather than transiting the passageway. The primary task is to monitor the number of vessels passing through the canal and verify this using ground truth data reported by canal authorities. Thus, we monitor all AIS messages within the passageway, defined by the POIs, as these are potentially transiting the canal/passageway. This ensures that our trade-related vessel count is as accurate as possible, and we are not missing any data points.

To verify the proposed indicators and demonstrate its application, we employed data from January 2019 to December 2019 and March 2021 to April 2021, respectively. Our chosen case study for demonstrating the methodology is the Suez Canal, a critical maritime trade route located at coordinates $31^{\circ }30^{\prime }$N $31^{\circ }55^{\prime }$E to $29^{\circ }45^{\prime }$N $32^{\circ }55^{\prime }$E, facilitating the movement of over 1 billion gross tons of cargo annually [26] . Any disruption within the canal significantly impacts global trade.

**Fig 1:**
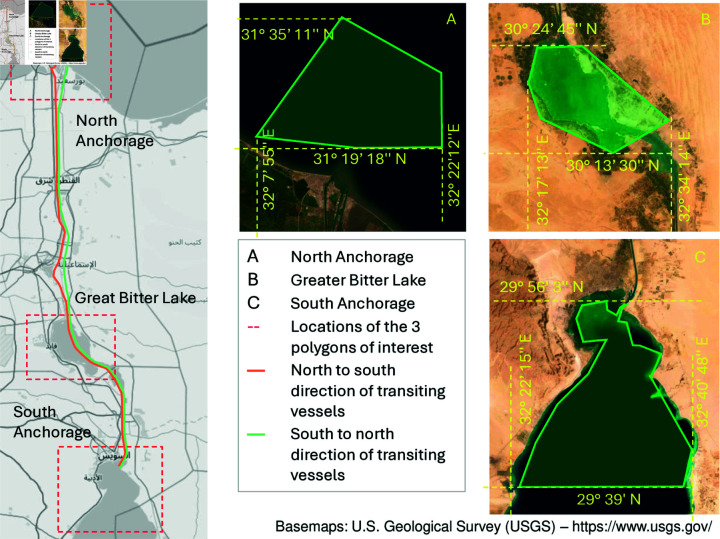
The area under study. The Suez Canal use case study analyzes vessel movement through three key polygons: North Anchorage: Situated near Port Said, captures vessels anchored at the northern entrance. South Anchorage: Located close to Suez Port, identifies vessels at anchor at the southern end. Great Bitter Lake: Encompasses the lake within Suez Canal, serving as a key waypoint for transiting vessels. By analyzing dwell time and presence within these zones, the study classifies vessels as queued or potentially have transited.

Unlike port analysis which focused on a single area of interest, analyzing passageways necessitates identifying at least two data collection areas defined by their POIs. These POIs are typically in the two end points of a passageway. For this study, we introduce an additional data collection area in between the two end points as redundancy. As illustrated in Fig 1, the three data collection areas for the Suez Canal analysis are: North Anchorage, Great Bitter Lake, and South Anchorage [11]. All AIS messages captured within these areas during the January 2019 – December 2019 (verification) or March 2021 – April 2021 (application) periods are utilized for analysis.

## Methods

**Fig 2:**
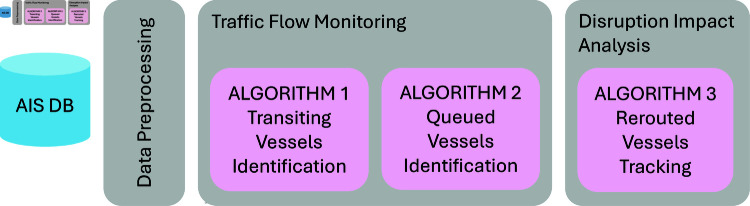
The proposed AIS-based framework. Our proposed framework is composed of traffic flow monitoring which provides insight on the day to day congestion as it develops and eases, and disruption impact analysis which captures the rerouting of external stakeholders.

Our proposed AIS-based framework for traffic flow monitoring and disruption impact analysis is presented in Fig 2. We detail each key algorithm employed in the process below:

### Traffic flow monitoring

Passageway analysis typically requires at least two POIs only *i.e.* the two entry points. However, we incorporated recordings from within the passageway due to two major limitations inherent to AIS data collection. Firstly, the pre-defined cut-off time might prematurely stop recordings for vessels completing the entire canal, leading to their presence being captured in just one polygon. Secondly, data loss can occur due to either intentional AIS disabling by ships or limitations in satellite receiver capacity. By introducing a third POI within the passageway *i.e.* Great Bitter Lake, we achieve an average increase of 10% in the detection rate of transiting vessels, as reflected in Table 2.

**Table 2:** Our proposed use of three POIs results in 9.93% average improvement in transiting vessel detection rate as compared to using only the two endpoints of the passageway. Test conducted in Suez Canal passageway from March 2021 to April 2021.

Count	using two POIs	using three POIs	% *↑* detection
Container ships	707	809	14.43%
Bulk Carriers	780	827	6.03%
Tankers	708	763	7.77%
Car Carrier	141	148	4.96%
General Cargo	283	346	22.26%
RoRo	24	26	8.33%
Passenger ships	2	2	0.00%
LNG	134	134	0.00%
TOTAL	2779	3055	**9.93**%

Algorithm 1 in S1 Appendix forms the foundation for identifying transiting vessels and analyzing anomalous events in passageways. As an illustration, consider the March 2021 Suez canal incident. We defined three POIs representing the two end points, North and South Anchorage and the midway point, Great Bitter Lake. All AIS data containing vessels registered within any of these POIs are collected. Consecutive AIS data timestamp were checked (Algorithm 1. line 6 or A1.6) and grouped (A1.7) to ensure that all AIS data collected within a particular time period were correctly assigned to voyages. This grouping was based on the average transit time which, for Suez Canal, is around 16 hours [27]. If the time difference (*Δt*) between consecutive AIS messages exceeded this average, a new voyage was considered to have started.

For all the voyages of a vessel, those appearing in at least two POIs were considered transiting vessels (A1.10), with their arrival timestamp at the passageway marked as the earliest recorded presence in any POI (A1.11). Due to the dynamic nature of vessel idle time, which depends on factors like traffic volume, vessel size or type, and seasonality, we also used dwell time which is equal to the sum of transit time and idle time, as additional variable (A1.12 and A1.13). We then filter the trade-related vessels *i.e.* cargo, tanker, and bulk carriers (A1.17 to A1.20). Finally, this routine outputs daily vessel counts, and dwell and idle times for the identified transiting vessels.

During normal operations or Business-as-Usual (BaU) period, we can statistically identify a maximum dwell time for the vessels using the absolute maximum or a certain percentile value. Deviations from this baseline, thus, serve as an indicator when the impact of anomalous events begins. To quantify the impact of delays on vessel traffic, Algorithm 2 in S1 Appendix, identifies vessels significantly affected by the blockage. Based on dwell time, we define “queued vessels" as those exceeding a threshold ($\tau_{0}$) (A2.6) set to the 95% of the maximum dwell time observed during the BaU period. This threshold focuses on events causing substantial delays compared to typical travel times.

### Disruption impact analysis

We propose an analysis to assess the impact on external stakeholders, focusing on the primary effect of passageway disruption, that is, vessel rerouting. Taking the Suez Canal incident as a case study, we present a comprehensive methodology outlined in Algorithm 3 in S1 Appendix, to identify new routes adopted by affected vessels rerouting around the Cape of Good Hope in South Africa. Unlike AIS data for vessels traversing the canal, this part of the analysis necessitates coverage of a broader area encompassing potential reroute paths. Given the absence of predefined geographical boundaries along the Cape route, we developed a systematic approach to determine the POIs for the Cape.

**Fig 3:**
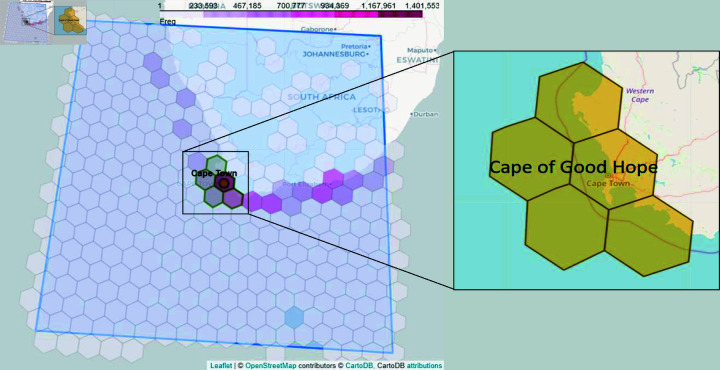
Deriving POI for the Cape of Good Hope. The blue area inside the quadrilateral is centered at Cape Town, South Africa edge to center is around 1,000 km. The area is divided into H3 indices with resolution 3. Each index is colored according to the distribution of AIS message with the darkest as the index with the highest frequency. The green H3 indices at the center represent the hexagons selected for the Cape of Good Hope polygon (see inset).

Beginning with a conservative approach, we selected a vast square area extending 1,000 km from the Cape Town coastline as the initial focal point (A3.3), depicted as trapezoidal boundary in Fig 3. This area adequately covered South Africa’s Exclusive Economic Zone, extending approximately 200 nautical miles or 330 km from the coast, and provided comprehensive coverage along the South African passage. An extended timeline from the blockage period was considered to accommodate travel time to the Cape. This dataset comprised approximately 71.2 million AIS messages recorded between 14 March 2021 and 30 April 2021.

To manage the amount of data for processing, each AIS message was bucketed into small hexagons (A3.4) using Uber’s Hexagonal Hierarchical Spatial Index (H3). Each hexagon is uniquely identified by an H3 index. The size of the hexagon depends on the chosen resolution which varies from 0 to 15, 0 being the lowest resolution with an average hexagon edge length of  ∼ 1 , 000 km and 15 being the highest resolution with an average hexagon edge length of 0.5 m.

In choosing the H3 resolution, the hexagon size should not be too large *i.e.* lowest resolution, that all AIS messages are clustered in just one or two hexagons. The hexagon size should be large enough that it resembles good distribution of AIS messages with adjacent hexagons. We empirically determined this size for the Suez Canal analysis, particularly for detecting rerouted vessels in Cape of Good Hope, see Table 3, and choose resolution 3 (A3.5). The last column in Table 3 (resolution 3) demonstrates the best balance in ship distribution across hexagons. If too many ships were aggregated into one hexagon, as seen in larger hexagons (resolutions 0 and 1), it would result in over-simplification and loss of spatial detail. On the other hand, smaller hexagons (resolution 4) increase computational complexity without significantly improving distribution, making resolution 3 ideal for preserving detail while maintaining efficiency. Among all hexagons with resolution 3, the hexagon with the most frequency of AIS message was the hexagon that contained Port Cape Town. We used this (A3.6), including its neighbor H3 indices, as the POI for the Cape, see Fig 3, inset area, for illustration.

**Table 3:** Distribution of AIS messages for the top 5 H3 indices for different resolutions in detecting rerouted vehicles in Cape of Good Hope. Resolution 3 is the largest hexagon, after resolutions 0 to 2, with fairly well distributed AIS messages in the top 5 hexagons. Smaller resolutions, i.e. resolutions 4 and up, would mean unnecessarily higher computational demands.

	H3 resolution	0	1	2	3
Average H3 Edge Length	1.107 km	418 km	158 km	60 km
Top 5 hexagons	1	49*%*	34*%*	20*%*	8*%*
	2	44*%*	34*%*	14*%*	4*%*
	3	7*%*	9*%*	12*%*	4*%*
	4	n.a.	6*%*	6*%*	4*%*
	5	n.a.	5*%*	5*%*	4*%*

Key vessel details were summarized for each H3 index, encompassing various parameters such as *datetime*, *latitude*, *longitude*, *draught*, *heading*, and *destination* (A3.8, A3.9). Heading values were recalculated to align with the latitude and longitude of the H3 index (A3.10), while unique destinations and arrival times were cataloged, separately.

Additionally, predefined areas including the North Atlantic Sea, Arabian Sea, Northern Indian Ocean, were identified as potential reroute points. This allows the identification of potential detour routes for affected vessels. Analyzing vessels identified at Cape Town’s POIs with the longest routes revealed their use as a potential rerouting point for 60 vessels (A3.12). Notably, 14 of these vessels exhibited clear detours (A3.13, A3.14), *i.e.* originally heading towards the Suez Canal, characterized by a distinct movement pattern, described as follows:

- Initial trajectory towards Suez, followed by a southward turn towards the Cape, resembling a U-turn.
- Prominent directional changes with high angular displacement.
- Directional alterations occurring in specific regions, such as the North Atlantic Sea for westward routes and the Arabian or Northern Indian Sea for eastward routes.
- Indication of Suez Canal or related destinations, despite not traversing these areas.

We then extended to encompass the Mediterranean Sea, Red Sea, and Gulf of Aden. These regions served as viable alternatives for vessels intending to access the Suez Canal (A3.15). Criteria were established to identify rerouted vessels based on declared destinations, resulting in the identification of 772 vessels meeting the selection parameters, of which 48 vessels were confirmed to have rerouted via the Cape of Good Hope.

## AIS-based indicators validation

To facilitate analysis of anomalous events at passageways, we propose three AIS-based indicators, namely, (1) the *daily vessel count* which tracks the daily number of transits for different vessel types, the (2) *daily median dwell and idle times* which represent the middle value of dwell and idle times for vessels on a given day, and the (3) *passageway event-driven routes* which reflect the overall impact of disruptions on maritime navigation.

### Understanding each indicator

The *daily vessel count* tracks the number of trade vessels passing through the passageway, counted when observed at a minimum of two POIs. It provides insights into traffic volume, helping gauge overall usage and identify shifts in transit patterns. By analyzing changes in vessel counts, we can assess how disruptions impact the activity level in a passageway. The *daily median dwell time* measures the time taken for a vessel to travel from entry to exit points within the passageway, calculated as the interval between the first and last sightings at POIs. By also extracting the corresponding *daily median idle time*, we can differentiate between delays and transit time. Longer dwell times may indicate inefficiencies and disruptions, impacting passageway throughput.

The *passageway event-driven routes* indicator captures the deviations in vessel routes due to events affecting the passageway. By analyzing rerouted paths, we can understand how disruptions influence navigational decisions and identify alternative routing patterns that arise during events, offering a clearer picture of the impact to maritime logistics. Incorporating these indicators allows us to understand the overall impact of disruptions. This, in turn, aids policymakers and maritime authorities in developing strategies to mitigate the effects of such disruptions and improve the resilience of maritime passageways and navigational routes.

### Vessel tracking validation

**Fig 4:**
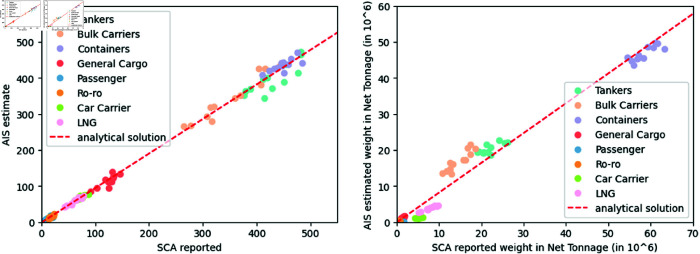
Estimated vs reported vessel count. The estimated and reported vessel count and net tonnage show strong linear relations between the two variables. Error with respect to the best fit line also shows low mean square error (MSE) of 12.59 and 2.58 (in $10^{6}$ metric ton) for the count and tonnage estimates, respectively.

Validation of these estimates was performed using data from the Suez Canal Authority (SCA) for 2019. Due to limited SCA reported indicators, we only compared the *daily vessel count* and *daily net tonnage* estimates with the corresponding SCA reported monthly count and net tonnage for specific vessel types, including tankers, LNG carriers, bulk carriers, container ships, and dry cargo vessels. Correlation analysis revealed a strong positive correlation between the AIS-based estimates and the reported data, as shown in Table 4. Fig 4 further visualizes this strong linear relationship for both count and net tonnage.

**Table 4:** Correlation values of AIS-based estimates and SCA reported values for 2019 monthly vessel counts.

Estimated and Reported	*r*	*p*-value	*N*
Tankers, Containers, Bulk Carriers Counts	.96	*p* < . 01	34
LNG, Dry Cargo Counts	.99	*p* < . 01	58
Tankers, Containers, Bulk Carriers Tonnage	.99	*p* < . 01	34
LNG, Dry Cargo Tonnage	.99	*p* < . 01	58

**Fig 5:**
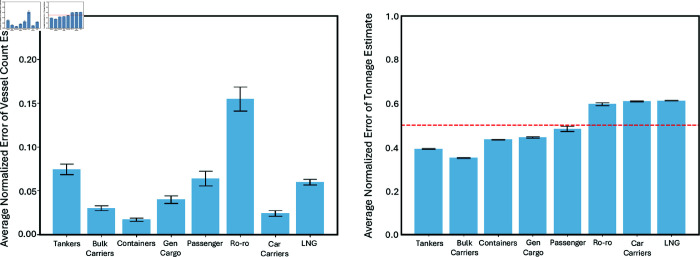
Average error between the estimated and reported count (left) and net tonnage (right) reveals that for vessel types Roro, Car carriers and LNG, AIS-based net tonnage estimation is not effective. The average error of these estimates exceeds 50% of the reported values of the SCA. Error bars: standard error of the mean.

However, while the proposed indicators demonstrate overall effectiveness, error analysis revealed limitations in estimating *daily net tonnage* for specific ship types. As shown in Fig 5, the estimated net tonnage for RoRo, car carriers, and LNG tankers exhibits an average normalized error exceeding 50% compared to actual reported values. Therefore, caution is advised when applying this indicator to estimate net tonnage for these specific vessel categories.

### Rerouted vessel detection validation

In detecting passageway event-driven routes, verification of the identified rerouted vessels was conducted against published online resources. A.P. Moller-Maersk (Maersk) reported 12 detoured vessels in [28]. We compared these vessels to our identified rerouted vessels to see if they were captured by our two criteria, i.e. Case 1: captured rerouting point, and Case 2: declared Suez as destination (see Table 5)

**Table 5:** Case Characteristics and standardized parameters for detecting rerouted vessels. Either of these two characteristics must be satisfied to qualify a vessel as rerouted vessel.

Case	Characteristic	Standardized Parameters
Case 1: Captured Point of Detour	Change in direction from North to South	Heading >90^∘^, if in North Atlantic Sea, Arabian Sea or Northern Indian Ocean
	Change in angular direction	Smallest angular difference between consecutive heading values is at least $15^{\circ }$ regardless of direction
	Within specified regions	Location H3 indices are in H3 indices of specified regions (see Appendix F for the polygons)
Case 2: Captured Suez areas as Destination	Suez Canal and related areas are destination	Destination contains any of the strings: “SUEZ”, “SZC”, “SUZ”,”PSD”, “SUC”, “EGYPT”, “SAID”,”EG”. The strings were determined based on those that passed Case 1

Only two Maersk vessels were not identified by our method. The first vessel, Maersk Santana, did not meet our criteria and showed no indication of approaching Suez during the blockage period. The second, Arnold Maersk, met Case 1 but not Case 2. However, the potential reroute was a typical route for vessels leaving Sri Lanka and heading south towards Cape of Good Hope.

Similarly, Mediterranean Shipping Company reported three detoured vessels in [29], of which two were identified by our method. The missed vessel, similar to Arnold Maersk, had a captured rerouting point, but the route appeared typical for vessels heading in that direction. Encouragingly, all nine detoured vessels reported by various shipping lines [30] were identified by our rerouting method.

## Analysis

### Disruption in the Suez Canal

**Fig 6:**
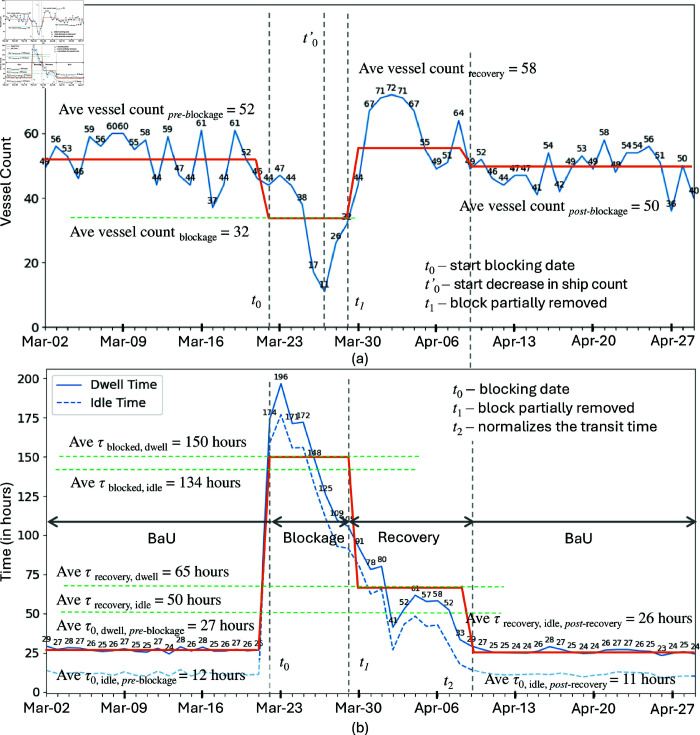
(a) Daily vessel count, and (b) daily median dwell and idle times of vessels transiting the Suez Canal. The vertical lines indicate critical time corresponding to the date when the ship started to block the canal ($t_{0}$), the date when there was a significant drop in ship arrivals ($t_{0}^{\prime }$), the date of the significant removal of the blockade ($t_{1}$), and the date when the average waiting transit time normalizes ($t_{2}$). The orange line represents the average per period. Data from 2 March 2021 until 29 April 2021.

Three time-series data were plotted to facilitate the analysis for the case in Suez Canal, the *daily vessel count* and the *daily median dwell and idle times*, see Fig 6. The two metrics were computed for observation period beginning of March 2021 until end of April 2021.

We divided the investigation of the unusual events into four periods, refer to Fig 6, namely, the (1) pre-incident Business as Usual (BaU) period which is before 22 March 2021 or $t_{0}$, the (2) Blockage period which is from $t_{0}$ to when the blocked ship was partially refloated on 29 March 2021 or $t_{1}$, the (3) Recovery period which is from $t_{1}$ to when the transit time normalizes back to the pre-incident BaU range, called $t_{2}$, and the (4) post-incident BaU which is from $t_{2}$, onwards. We observed that for *daily vessel count*, there was a drop a few days after $t_{0}$ with a gradual increase starting $t_{1}$ when the canal was partially opened. These arrivals reached figures higher than those from pre-incident BaU period, presumably from those vessels which decided to delay their transit via Suez Canal during the blockage period. During the blockage and recovery period in the Suez Canal blockage event, out of 690 transiting vessels, 73.48% or 507 vessels suffered significant delay while the remaining transiting cargo are assumed to be rerouted.

Following the blockage, *the average median dwell time (orange curve) mirrored the anomalous event duration, halving during recovery period despite increased vessel arrivals, and dropping back to the pre-incident BaU level within 2-3 days of returning to normal traffic levels*. During the blockage, the increase in dwell time was predominantly due to a significant rise in idle time, refer to Fig 6. This idle time accounted for approximately 89% of the total dwell time during the peak of the blockage. Vessels were forced to wait for extended periods as the canal was obstructed, resulting in a dramatic increase in idle time as ships remained stationary, unable to transit through the canal. As efforts to clear the blockage and restore normal operations progressed, there was a noticeable decrease in idle time. During the recovery phase, idle time constituted around 77% of the total dwell time. This reduction can be attributed to the gradual resumption of vessel movements as the affected portion of the canal was being cleared and managed. Post-recovery, the proportion of idle time decreased further to 42% of the total dwell time. This significant drop indicates a return to more typical transit conditions, where vessels experienced less waiting time and could pass through the canal with fewer delays. This prolonged idle time during the blockage forced many vessels to seek alternative routes.

The significant delays and high percentage of affected vessels (73.48%) highlight the need for improved contingency planning and information sharing among canal authorities, shipping companies, and relevant stakeholders. This could involve pre-established reroute plans for different scenarios, considering potential destination impacts and enhanced real-time information sharing about expected delays and rerouting options to allow informed decision-making. The observation of increased vessel counts after the blockage suggests potential strain on capacity. This could necessitate investigating infrastructure upgrades or exploring bypass route development to mitigate future disruptions.

**Fig 7:**
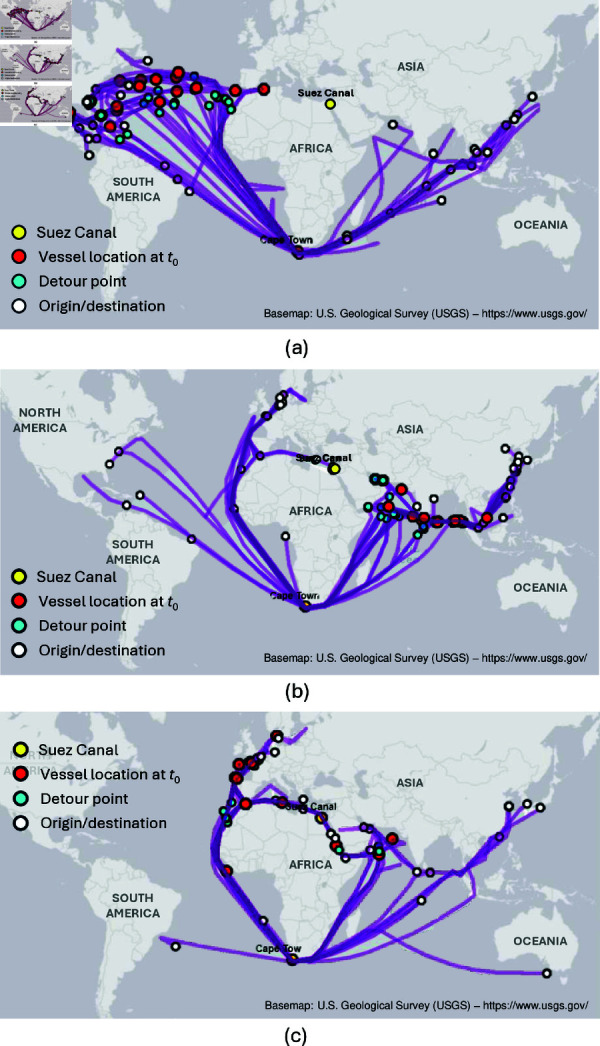
Different reroutes via Cape of Good Hope. Our experiments reveal three main reroutes via Cape of Good Hope for vessels that supposed to pass Suez Canal. These include (a) from North America to Asia, (b) from Asia to North and South Americas, and (c) from Europe to Asia, or vice versa. Reroute study was conducted on data from 14 March 2021 and 30 April 2021.

Analyzing the rerouted vessels revealed three distinct group of routes based on their origin, destination, and detour points as illustrated in Fig 7a–7c. The first group, as shown in Fig 7a, consists of vessels from the North and South America, headed to Suez Canal, through the Strait of Gibraltar, but rerouted at the North Atlantic Sea within a few days after the blockage. Ports of destination are mostly in Singapore or Malaysia. A total of 19 vessels followed this route. The second group, depicted in Fig 7a, consists of westbound vessels from Asia to either Europe or the Americas. During the blockage, the vessels were in the Arabian sea or have just exited the East Asia region near Singapore. Entry point to Suez Canal was via Gulf of Aden, but rerouted, passing through Cape of Good Hope. There were 21 rerouted vessels that used this route. Finally, the third group, shown in Fig 7c, consists of vessels that were mostly from Europe and headed to Asia. Detour points for vessels accessing Suez Canal from the north were near the Strait of Gibraltar and Mediterranean Sea, while from the south were near the Gulf of Aden. There were 8 rerouted vessels that used this route.

No rerouted vessels were at the North and South Anchorages. This suggests that *most vessels already in the queue opted to wait and vessels choosing to detour were further from the congested zone*. This suggests a need for optimizing queue management systems that could involve prioritizing vessels based on factors like cargo type, destination urgency, and size, implementing efficient queue management protocols to minimize wait times, and incentivizing early rerouting. Also, providing clear and up-to-date information about expected wait times and potential rerouting options would allow informed decision-making by shipping companies.

### Disruption in other passageways

**Fig 8:**
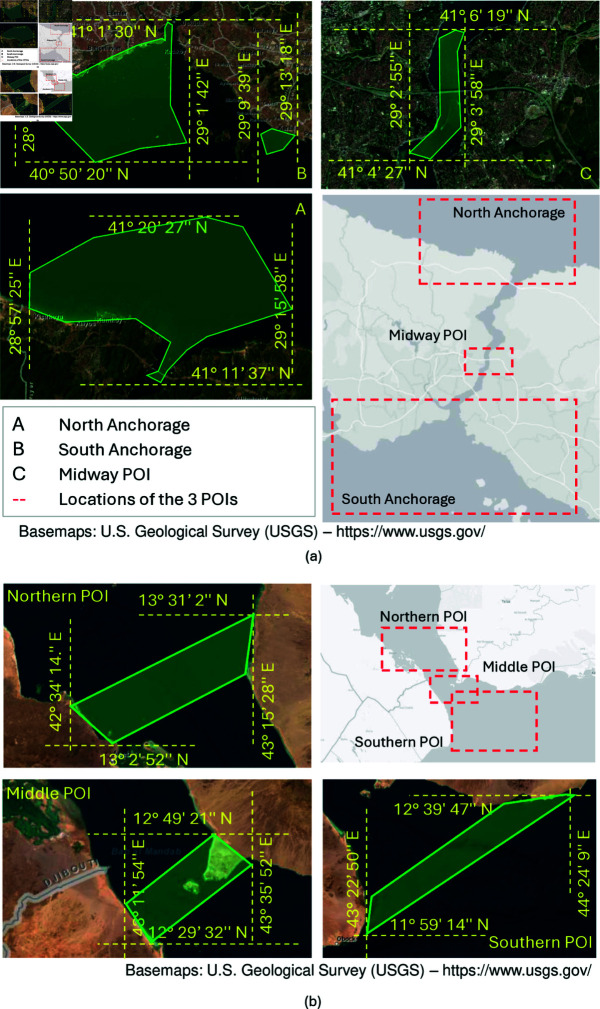
The (a) Bosporus Strait and (b) Bab el-Mandeb Strait POIs. The north and south POIs in the Bosporus align with the entrance traffic lanes and anchorage areas, while the midway POI is at the narrowest point (700 meters wide) between Kandilli and Aşiyan [32]. For the Bab el-Mandeb Strait, POIs were chosen based on geographical features: the southern opening, the narrowest point (26 km wide), and the northern area where the strait widens.

To demonstrate the generalizability of our proposed framework, we applied it to two other cases of disruptions in passageway, namely the Bosporus Strait and Bab el-Mandeb Strait, focusing on identifying transiting and queued vessels to analyze the impact on vessel counts and dwell time. These impacts were due to policy changes from the Russian invasion of Ukraine and a Houthi attack, respectively. The Bosporus Strait connects the Black Sea to the Sea of Marmara and plays a strategic role in international trade, particularly in grain and oil, with over 630M gross tons passing through the strait in 2021 from 38,500 vessels [31]. The Bab el-Mandeb Strait, connecting the Red Sea with the Gulf of Aden and the Indian Ocean, assumed significant strategic and economic importance with the construction of the Suez Canal, controlling almost all shipping between the Indian Ocean and the Mediterranean Sea via the Suez Canal. Fig 8a and 8b illustrates the three POIs for both passageways.

**Fig 9:**
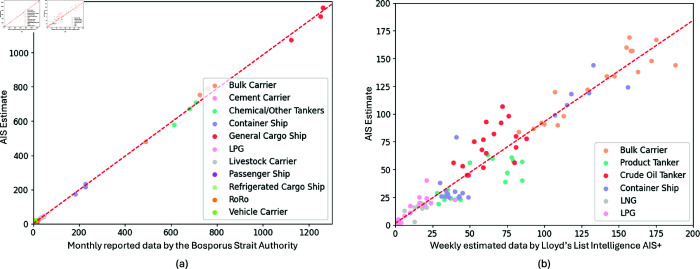
Comparison of Vessel Counts: (a) Estimated vs. Reported in the Bosporus Strait (from January 2022 to March 2022) and (b) Estimated vs. proprietary AIS+ approaches in the Bab el-Mandeb Strait (from November 2023 to March 2024). In (a), the estimated and reported counts show a strong linear correlation, with a low MSE of 8.96 for a total vessel count estimate of 8507, indicating minimal deviation from the best fit line. Similarly, in (b), our estimated counts correlate strongly with AIS+ estimates, with a low MSE of 9.39 for a total vessel count estimate of 5775, also indicating minimal deviation from the best fit line.

We validated our estimated vessel counts for the Bosporus Strait against the reported data from the Ministry of Transport and Infrastructure of the Republic of Turkey [33] for the period of January to March 2022. The results demonstrated a strong correlation between our estimates for each ship category and the official figures, as illustrated in Fig 9a. Our AIS-based total vessel estimate was 8507, while official data reported 8563. This demonstrates the wider applicability of our methodology. In the case of the Bab el-Mandeb Strait, which did not have a centralized authority, we validated using Lloyd’s List Intelligence data [34], which are generated using terrestrial AIS networks, satellite AIS, and shipborne receivers. Our AIS-only and Lloyd’s “AIS+" methodology estimates have a strong correlation (*r* = . 96 , *p* < 0 . 001). Our total vessel estimate was 5775 while the AIS+ was 6139. See Fig 9b

Lloyd’s List Intelligence’s AIS+ approach [35] integrates multiple data sources, including proprietary terrestrial AIS networks, satellite AIS, and shipborne receivers, to provide vessel tracking intelligence. Their methodology involves using an AI technology to remove the duplicate and validate AIS positions, combined with a geospatial database for insights on vessel movements. This system ensures accuracy and coverage, leveraging a proprietary terrestrial network and satellite data to fill gaps in AIS coverage. On the other hand, our streamlined approach is more cost effective, as it relies solely on publicly available AIS signals data, and our open-source methodology and algorithm enable replication by researchers.

**Fig 10:**
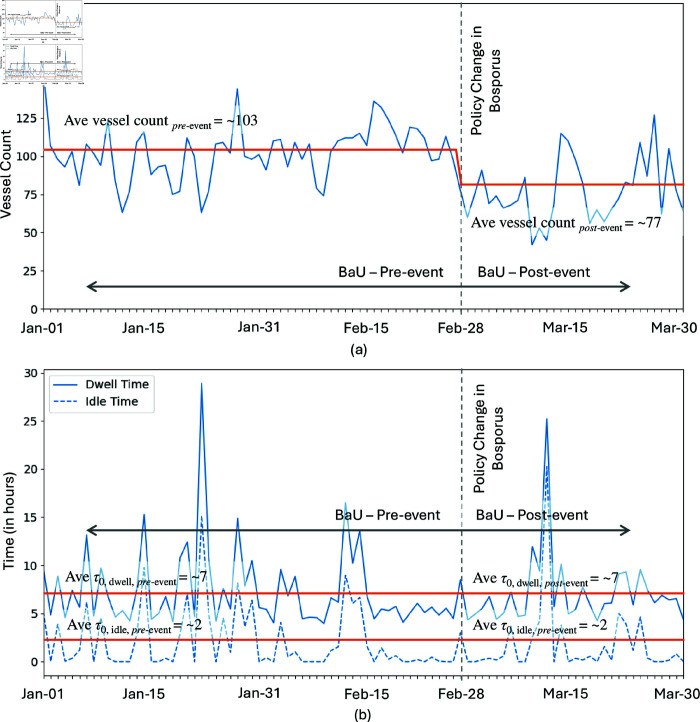
Daily vessel count (a) and daily median dwell and idle time (b) of vessels transiting the Bosporus Strait. The vertical lines indicate critical time corresponding to the date when the policy change was implemented. The orange line represents the average per period. Data from 1 January 2022 until 31 March 2022. Although the number of vessels declined significantly after the policy was enacted, there was no discernible change in the dwell or idle times of the vessels.

**Fig 11:**
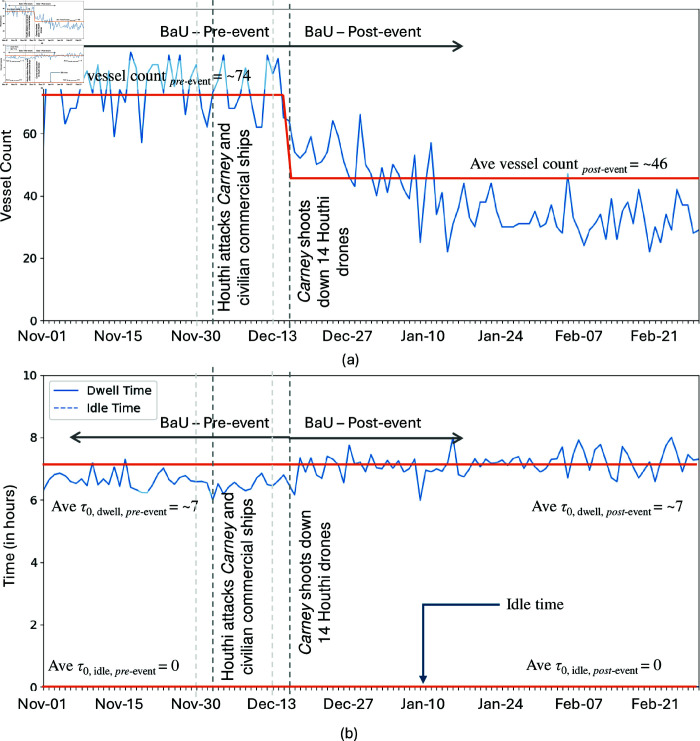
Daily vessel count (a) and daily median dwell and idle time (b) of vessels transiting the Bab el-Mandeb Strait. The vertical lines indicate critical time corresponding to the Houthi attacks and *Carney*’s retaliation. The orange line represents the average per period. Similar to the Bosporus Strait’s incident, post-event vessel dwell and idle times were unchanged, even as vesse counts were significantly lower. Data from 1 November 2023 until 28 February 2024.

Recent geopolitical events significantly impacted ship transits through these major passageways. Turkey’s response to Russia-Ukraine war led to a policy change of enforcing the wartime provisions of the Montreux Convention, closing the Bosporus Strait to warships from any country starting 28 February 2022, except for those returning to a home base in the Black Sea. Although this policy targets warships, our methodology reveals a notable decrease in overall trade vessels through the Bosporus (Fig 10a). Similarly, the conflict in the Bab el-Mandeb Strait, marked by the Houthi attack on 3 December 2023, involving anti-ship ballistic missiles, and the subsequent retaliation by the vessel *Carney* on 16 December 2023, resulted in a significant drop in vessel transits (Fig 11a). These disruptions illustrate the profound effects of geopolitical tensions on maritime traffic.

**Fig 12:**
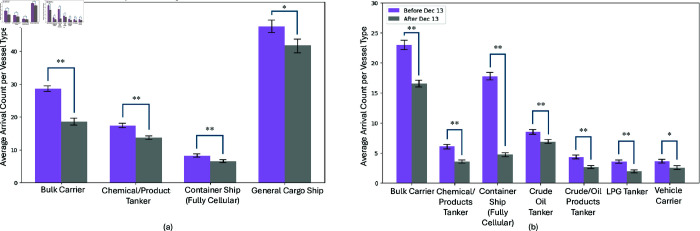
Significant Decrease in Vessel Count of Trade Vessels in (a) Bosporus Strait and (b) Bab el-Mandeb Strait. Using AIS vessel types, we identified the vessel types most affected by disruptions in both straits. The variations in affected vessel types reflect the economic priorities served by each strait. ** means *p* < . 01, * means *p* < . 05, and n.s. means not significant.

The decrease in trade vessels in the Bosporus Strait is significant for Bulk Carriers, Chemical/Product Tankers, Container Ships, and General Cargo Ships (see Fig 12a). A similar trend is observed for vessels passing through the Bab el-Mandeb Strait, with the exception of General Cargo Ships. Additionally, there is a notable reduction in vessels carrying Crude Oil Products, LPG, and vehicles in Bab el-Mandeb (see Fig 12b). This discrepancy is possibly due to the differing economic priorities of the regions. The Bosporus primarily facilitates European-Asian trade, which may explain the pronounced decrease in general cargo types, whereas Bab el-Mandeb’s broader role in global energy transport might account for the significant impact on energy-related vessels.

**Fig 13:**
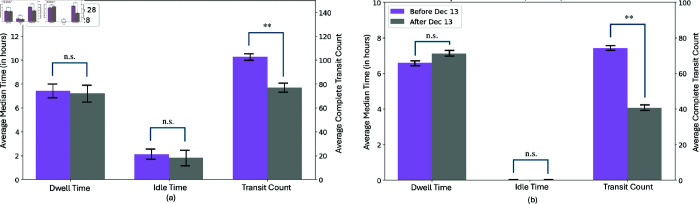
Comparison of the median time and transit count before and after an event in (a) Bosporus Strait and (b) Bab el-Mandeb Strait. For both passageways, the events significantly impacted vessel transit count in both straits, while, unlike in Suez Canal, the idle time, and thus, the dwell time, remains unaffected. ** means *p* < . 01, n.s. means not significant

While the number of transits decreased, the transit times remained largely unaffected (see Fig 10b, Fig 11b, Fig 13b and 13b). This outcome can be attributed to the fact that the passageways were not completely obstructed. Vessels that could still pass through did so without significant delays, unlike in Suez Canal scenario where full blockage occur, forcing ships to remain stationary or seek alternative routes. This suggests that *partial disruptions, while impacting the volume of traffic, do not necessarily translate to longer dwell times as long as a passable route remains available.*

### Insights from analyzing maritime disruptions

Our proposed framework can track vessel movements during disruptions. AIS-derived indicators, such as vessel count and dwell time, offer a comprehensive view of disruption impacts across different stages: pre-event, during the event, and post-event recovery. These indicators have numerous applications: for maritime authorities, such as port managers and coastal patrols, these statistics enable near-real time monitoring of passageway operations which are critical during disruptions; for researchers, they can be extended to examine implications for international flows of goods, environmental and economic impact of excessive idle times, and other topics.

For effective analysis, it is recommended to combine AIS-based indicators with ground truth data whenever available, particularly for canals and regulated passageways. This combination validates the extracted values and enhances accuracy. When selecting POIs, use predefined anchorages for entry, middle, and exit points when possible. If predefined anchorages are not available, base the POI selection on the density of AIS signals using viable clustering method *e.g.* [3,36] or geographical location and features of the passageway. Additionally, for fine-grained analysis of vessel types, using the Ship Register field rather than the AIS Vessel Type field is recommended, as it provides a more detailed and nuanced categorization of vessels, improving classification precision.

Our framework also enables real-time monitoring of both the nature of disruptions and the types of vessels affected. It identifies disruptions through fewer vessels and prolonged delays, measured by vessel count, dwell time, and idle time. Providing such data in real time allows ship captains to update their voyage plans more effectively. Additionally, by integrating container and draught information, the framework can estimate the monetary value of affected goods. This timely data proves valuable for commodity and insurance markets, enabling them to respond swiftly to disruptions in maritime traffic.

A key insight from our analysis is the impact of blockage severity on vessel behavior. For full blockages, such as the Suez Canal incident, vessels close to the blockage tend to wait, while those farther away are more likely to reroute. This distinction underscores the role of proximity in influencing vessel decisions. In contrast, during partial blockages, where the passageway remains navigable, we observe a significant drop in daily vessel counts, while dwell times remain stable. This indicates that even partial functionality of a passageway can mitigate overall disruptions to maritime traffic. Maritime authorities and logistics companies can use these insights to understand vessel behavior during disruptions and provide more effective contingency plans.

### Data limitations and optimization approaches

AIS data have two limitations which are worth noting. Firstly, as AIS signals are based on radio frequencies, they could be far fewer during extreme weather as significant atmospheric noise during storms could disrupt the sending and receiving of signals. Hence, AIS data frequency at every few seconds should be used with caution. Secondly, AIS signal “spoofing" remains a potential challenge for research based on this data source. “Spoofing" refers to the deliberate manipulation of a vessel’s AIS to broadcast false information to evade sanctions or hide illicit activities. Errors in transit count estimates due to spoofing can be minimized by using additional data sources, such as satellite optical data, to validate the estimates. However, this will further impact computational efficiency due to the need for additional data preprocessing and merging.

As the number of transiting vessels in a passageway increases, the accuracy of the estimated transit count improves because the impact of individual misdetections diminishes, thereby reducing the overall variance. This trend is evident in the MSE of our estimates for the Suez Canal, Bab-el Mandeb, and Bosporus passageways, with 3,052, 5,775, and 8,507 detected transiting vessels, respectively. The MSEs are 12.59, 9.39, and 8.96, respectively.

However, increasing the number of vessels being monitored can impact computational efficiency due to the need for additional resources, *i.e.* time and memory. To mitigate this, we used pandas DataFrame for efficient data handling and vectorized operations. Optimization strategies, such as parallel processing, can distribute the workload across multiple processors, potentially decreasing the complexity by a factor of *p*, where *p* is the number of processors.

## Conclusion

This paper introduces a novel framework for analyzing anomalous events in passageways using Automatic Identification System (AIS) data. By incorporating data collection points within the passageway, this approach improves vessel detection by approximately 10% compared to other AIS-based detection methods. The framework was validated in the Suez Canal disruption case and further demonstrated in the Bosporus Strait and Bab el-Mandeb disruptions. Using the proposed approach allows us to discover distinct phases of a passageway disruption. It reveals that in full blockages, such as the Suez Canal, vessels near the blockage tend to wait, while those farther away choose alternative routes. In partial disruptions, like those in the Bosporus and Bab el-Mandeb Straits, traffic volume is affected, but dwell times remain stable as long as the passageway is navigable.

The proposed framework offers several key advantages. It delivers high-frequency estimates of vessel counts (all vessel types) and net tonnage (specific vessel types) with less than 0.2 and 0.5 average normalized error with respect to the reported values, respectively. This near real-time capability enables day-to-day analysis of queued vessels, waiting times, and, potentially, estimated cargo value, when something anomalous happens in passageways. Additionally, the framework facilitates the identification of reroutes for affected cargo not yet within the passageway, allowing for proactive measures. This near real-time advantage, compared to traditional sources with 2-3 month update cycles, offers significant advantages in both timely voyage planning and management, enabling more informed decision-making based on current conditions. Moreover, the ability to track reroutes and analyze real-time data can contribute to increased resilience in global supply chains by facilitating proactive measures.

To improve the methodology, it is recommended to refine the dwell time analysis to distinguish between complete and partial blockages. This involves incorporating parameters that monitor the impact on daily median dwell times and traffic volumes. Such refinements will enable more effective response strategies, optimize the rerouting process, and enhance the overall resilience of the maritime logistics network. Additionally, integrating AIS-based metrics, such as vessel type and draught, can enhance the framework’s ability to estimate the cargo value affected. By estimating the economic value of affected cargo, the framework can provide a more comprehensive understanding of the financial implications, leading to better-informed decision-making and more effective disruption management.

## Supporting information

S1 Appendix Implementation algorithms for the proposed AIS-based framework. (PDF)
