# Supplementary material for: Analyzing anomalous events in passageways with high-frequency ship signals
Source: PLoS One. 2025 Apr 1;20(4):e0320129. doi: 10.1371/journal.pone.0320129 (PMC11960904; doi:10.1371/journal.pone.0320129)
Supplement: S1 Appendix — (PDF) [file pone.0320129.s001.pdf]

Appendix

Algorithm 1 Transiting Vessels Identification

Input: AIS data for the observation period
Output: List of transiting vessels; Time-series data: daily vessel count, daily dwell time, and daily idle time
procedure IDENTIFYTRANSITINGVESSELS(AIS data)
    Define polygons of interest (POIs)
    for all vessels **do**
        Check time difference between consecutive AIS data, $\Delta t \leftarrow t_{current} - t_{prev}$
        Group AIS messages with Δt's less than average transit time into voyages
        for all voyages **do**
            Check AIS presence in at least two out of three POIs for each voyage
            if present in $\geq$ two POIs **then**
                arrival date $\leftarrow$ first AIS message date per vessel transit
                dwell time $\leftarrow$ time interval between AIS data at first and last POI
                idle time $\leftarrow$ duration when vessel speed is below 1 knot in all POIs
            else
                remove voyage from the list of voyages
            end if
            Check vessel type
            if vessel is not trade-related type **then**
                remove vessel from the list of vessels
            end if
        end for
    end for
    Derive time-series data: daily count of vessels and daily median dwell time, and daily median idle time
end procedure

Algorithm 2 Queued Vessels Identification

Input: List of transiting vessels
Output: List of queued vessels
procedure IDENTIFYQUEUEDVESSELS(List of transiting vessels)
    Start
    Identify transiting vessels whose start and end POI registers are either North or South
    if dwell time $> \tau_0$ **then**
        assign vessel as *queued* vessel
    end if
    End
end procedure

Algorithm 3 Rerouted Vessels Tracking

```
Require: AIS data, potential route areas
Ensure: Identified vessel reroutes
procedure IdentifyReroutes(AIS data, potential route areas)
    Identify Vessels via a Reroute Path POI:
    Start with a large area for potential route data collection
    Divide the area into grid-based regions for analysis
    Choose optimal grid resolution based on data density
    Select the region(s) with highest message density as POI
    Create Route Segments for Identified Vessels:
    Group AIS data into route segments based on grid regions
    Summarize key voyage details and vessel identity
    Adjust headings to match grid region positions
    Determine Potential Reroutes:
    Visually inspect vessels for deviations from expected paths
    Look for common reroute characteristics
    Consider vessels with significant direction changes
    Expand investigation to additional potential reroute points
end procedure
```
